# The history of small extracellular vesicles and their implication in cancer drug resistance

**DOI:** 10.3389/fonc.2022.948843

**Published:** 2022-08-24

**Authors:** Stefano Palazzolo, Vincenzo Canzonieri, Flavio Rizzolio

**Affiliations:** ^1^ Pathology Unit, Centro di Riferimento Oncologico di Aviano (CRO) Istituto di ricovero e cura a carattere scientifico (IRCCS), Aviano, Italy; ^2^ Department of Molecular Science and Nanosystems, Ca’ Foscary University, Venice, Italy

**Keywords:** extra cellular vesicles, cancer, drug resistance, therapy, oncology

## Abstract

Small extracellular vesicles (EVs) in the last 20 years are demonstrated to possess promising properties as potential new drug delivery systems, biomarkers, and therapeutic targets. Moreover, EVs are described to be involved in the most important steps of tumor development and progression including drug resistance. The acquired or intrinsic capacity of cancer cells to resist chemotherapies is one of the greatest obstacles to overcome to improve the prognosis of many patients. EVs are involved in this mechanism by exporting the drugs outside the cells and transferring the drug efflux pumps and miRNAs in recipient cells, in turn inducing drug resistance. In this mini-review, the main mechanisms by which EVs are involved in drug resistance are described, giving a rapid and clear overview of the field to the readers.

## Introduction

Extracellular vesicles (EVs) are small cell-released particles with a diameter ranging from 30 to 1,000 nm (1). EVs are a heterogeneous population that can differ in size, properties, and biological function and classified according to their biogenesis pathway (2). In addition, from the first attributing role, consisting in managing cellular waste, nowadays it is well recognized that EVs play a central role in cell–cell communication (3), both in physiological and in pathological conditions, and their cargoes have been distinguished in different components from proteins to miRNA, going through mRNA and lncRNA, among others (4). EVs were also employed as drug delivery systems (DDS) displaying very suitable properties for this purpose and obtaining interesting results in preclinical and clinical trials (2, 5).

The history of EVs started in the second half of the 1940s in the previous century, when in 1945 Chargaff working on blood coagulation observed small “*membrane debris*” sedimented at high-speed centrifugation of plasma supernatant (6). The following year, his observation was reported as “a variety of minute breakdown products of blood corpuscules” (7). Twenty-one years later, Peter Wolf described in more detail Chargaff’s remarks, saying that it could be a material “originated from platelets but it is distinguishable from the intact ones”. This claim was confirmed by electron microscopy images that Wolf himself described as “platelet dust” (8). For almost 20 years, other electron microscopy images showed structures with a size under 1,000 nm. In particular, in 1974, Nunez et al. reported, for the first time, structures later called multivesicular bodies (MVB) (9), opening up the path in the identification of a subtype of EVs that originated from MVB, later called exosomes or small EVs (30–150 nm). The biogenesis of these structures was demonstrated to start from late endosomes, which are formed by the inward budding of MVB membranes forming intraluminal vesicles (ILVs), which fuse back with the plasmatic membrane and released by cells as small EVs [later called exosomes (10)] as described by Cliff Harding in 1983 (11). Starting from the early 1980s, many studies on EVs have increased the knowledge in this field and scientists began to deeply understand the multiple biological functions in which EVs are involved. For almost a decade, small EVs were identified as a vehicle to remove unnecessary molecules from cells, like a cellular garbage disposal (12). In the 1990s, small EVs were identified to have an immunological function (13), followed by a large number of studies highlighting that EVs were involved in intercellular communication mechanisms playing a role in physiological or biologically important processes, such as lactation, inflammation, cell proliferation, and neuronal function (14–16). Moreover, other studies showed that EVs are implicated not only in pathological processes, namely, thrombosis (17), diabetes, and atherosclerosis (18), but also in the development and progression of diseases such as liver (19) and neurodegenerative diseases (20) and, recently, in cancer (21, 22). In cancer, many processes like cell proliferation, migration, invasion, epithelial-to-mesenchymal transition, angiogenesis, lymphogenesis, immune suppression, and metastasis (23) are regulated by EVs. In the late 1990s, important studies were published about EVs. Starting with the work of Raposo et al. (13) that demonstrates that EVs derived from immune cells are capable of presenting antigens, other groups started new projects about a new vaccine approach based on EVs. The first approach on vaccines using EVs was explored by Zitvogel et al. in 1998 (24). In their work, the authors described how EVs secreted by dendritic cells loaded with tumor antigens are able to eradicate cancer cells. Based on advances in the next decades, Escudier et al. conducted a clinical trial (25). This work has been a starting point for many studies on the physiological role of EVs and their possible applications as biomarkers, and an opportunity to new therapeutic approaches. In the last few years, lines of evidence for the implication of EVs in the development of anticancer drug resistance have increased and have been extensively studied. This mini-review will focus on the role of EVs in cancer drug resistance exploring and describing the main mechanisms of action through a synthetic description of the major scientific works in the field. Also, a brief description of the most important research papers is provided in **Table 1**, which aims to give an impression of this field and, overall, to give the readers a rapid and clear overview of the involvement of EVs in drug resistance mechanisms.

Table 1EVs cargoes and drug resistance mechanisms.

**Table:** 

miRNA												
Cell of origin		EV content	Target			Cancer type		Type of resistance		Mechanism		Ref.
MCF-7 and MDA-MB-231 DOX and PTX-resistant cells MCF7 CSCs		miR-155	TGF-β, FOXO-3a, and C/EBP-β mRNA			BC		DOX and PTX resistance		Contributing to drug resistance and promoting EMT and CSC phenotypes		(26)
MDA-MB-231 cells		miR-1246	CCNG2			BC		DOC, EPI, and GEM resistance		Promoting cell proliferation, migration, and drug resistance		(27)
BC cells resistant to TAM		miR221/222	P27 and ERα			BC		TAM resistance		Downregulation of p27 and ERα protein increasing cell proliferation		(28)
Trastuzumab-resistant BC cells		miR-567	ATG5			BC		Trastuzumab resistance		Regulating autophagy		(29)
MCF7		miR-567	ATG5			BC		Trastuzumab resistance		MiR-567 delivered by EVs revert cell resistance to trastuzumab		(30)
HL60/AR		MRP-1; miR19b, miR20a	HL60			Acute myeloid leukemia		MDR		Transferring chemoresistance through EVs from resistant to sensitive cells		(31)
MiaPaCa, Colo-357		miR-155	Unknown			Pancreatic cancer		GEM		Small EV-mediated mechanism of drug-induced acquired chemoresistance in PC cells. miR-155 induced suppression of gemcitabine-metabolizing enzyme, DCK		(32)
MCF7-Tam		miR-221/222	MCF7			BC		TAM		Vesicles containing miR-221/222 act as signaling molecules in cell–cell communication for tamoxifen resistance		(33)
786-0 Sor res, ACHN sor res.		miR-31-5p	786-0 Sor sens, ACHN sor sens			Advanced renal cell carcinoma		Sorafenib		EVs shuttled miR-31-5p can transfer resistance information from sorafenib-resistant to sensitive cells by directly targeting MLH1		(34)
SYO-1, HS-SYII, 1273/99 and YaFuS-resistant cells		microRNA-761	SYO-1, HS-SYII, 1273/99, and YaFuS			Synovial sarcoma		Pazopanib		EV miR-761 delivering affects chemosensitivity of synovial sarcoma cells to Pazopanib by targeting TRIP6, LMNA, and SIRT6		(35)
TMZ-resistant GBM cells		miR-1238	GBM-sensitive cells			Glioblastoma		Temozolomide		MiR-1238 levels are higher in TMZ-resistant GBM cells and their small EVs than in sensitive cells. Higher levels of miR-1238 are found in the sera of GBM patients than in healthy people. The loss of miR-1238 may sensitize resistant GBM cells by directly targeting the CAV1/EGFR pathway		(36)

**Table:** 

Proteins												
Cell of origin	EV content		Target			Cancer type		Type of resistance		Mechanism		Ref.
ADM-resistant MCF-7 cells	UCH-L1, P-gp		MAPK/ERK			BC		ADM resistance		Overexpression of UCH-L1 enhanced multidrug resistance in BC		(37)
Peripheral blood Evs from BC patients	TRPC5		P-gp			BC		Anthracycline/taxane-based chemotherapy		EVs stimulate the production of P-gp in the recipient cells by Ca2^+^- and NFATc3-mediated mechanisms		(38)
EVs derived by PTX treated MDA-MB-231 cells	Survivin		N/A			BC		PTX resistance		Promoting cell survival and drug resistance		(39)
DOC-resistant variant of MCF-7	P-gp		Stimulating drug efflux			BC		DOC resistance		Drug resistance is transferred as well as P-gp from drug-resistant to sensitive BC cells		(40)
HER2-positive BC cells	TGFβ1 and PD-L1		Unknown			BC		Trastuzumab resistance		Neuromedin U induces the escape of immune response in HER2-positive BC cells by increasing the expression of TGFβ1 and PDL1		(41)
HER2 positive SKBR-3 and BT474 cells	HER2		Unknown			BC		Trastuzumab resistance		Inhibition of Trastuzumab activity *in vitro*		(42)
Basal-like BC cells	PD-1		Unknown			BC		Immunosuppression		ESCRT-related protein ALIX regulates EGFR activity and PD-L1 surface presentation in BC cells		(43)
Mesenchymal stem cells	TGFβ, C1q and semaphorins		PDL-1 overexpression			BC		Immunosuppression		Inducing differentiation of monocytic myeloid-derived suppressor cells into highly immunosuppressive M2-polarized macrophages at tumor beds		(44)
Su-DHL-4, Balm-3, OCI-Ly1	CD20		Unknown			B-cell lymphoma		Rituximab resistance		EVs protect target cells from rituximab action through the expression of CD20		(45)
DU145RD and 22Rv1RD	MDR-1/P-gp		Unknown			Prostate cancer		DOC		Small EVs expelled from DU145 and 22Rv1 docetaxel-resistant variants (DU145RD and 22Rv1RD) conferred docetaxel resistance to DU145, 22Rv1 and LNCaP cells		(46)
MCF7 ADM res	P-gp/TrpC5		HME cells			BC		ADM		MCF-7/ADM cell-derived MVs transferred both P-gp and TrpC5 to HMECs, and TrpC5-containing MVs modulated the expression of P-gp in HMECs *via* the translocation of the transcription factor NFATc3		(47)
MG-63DXR30	MDR-1 mRNA/P-gp		MG-63			Osteosarcoma		DOX resistance		Multidrug-resistant osteosarcoma cells are able to spread their ability to resist to the effects of doxorubicin treatment on sensitive cells by transferring small EVs carrying MDR-1 mRNA and its product P-glycoprotein.		(48)
KBv200	ABCB1		KB			Epidermoid carcinoma		MDR		Chemotherapeutic agents can increase Rab8B-mediated release of EVs containing ABCB1 from drug-resistant cells to sensitive recipient cells; acquire a rapid but unsustainable resistance to evade the cytotoxicity of chemotherapeutic agents.		(49)
OSCC cell lines	ATP1A1, ATP1B3		Unknown			Oral squamous cell carcinoma		CPT resistance		OSCC-derived EVs may regulate cisplatin resistance through a cellular efflux system		(50)
RKO/R	p-STAT3, GSTP1p		Unknown			CRC		5-FU resistance		p-STAT3-containing small EVs contribute to acquired 5-FU resistance in CRC.		(51)
SGC-7901/VCR	CLIC1		SG7901			Gastric cancer		Vincristine		Small EVs transferring CLIC1 could induce the development of resistance to vincristine *in vitro*		(52)
BC cells under hypoxic conditions	TGFβ and IL10		Unknown			BC		Immunosuppression		Suppress T-cell proliferation *via* TGFβ		(53)
Acute lymphoblastic leukemia cell line MDR	P-gp		Unknown			Acute lymphoblastic leukemia		MDR		Purified EVs transfer functional P-gp from resistant cancer cells to drug-sensitive cells *in vitro*		(54)

**Table:** 

LncRNAs												
Cell of origin	EV content		Target			Cancer type	Type of resistance		Mechanism		Ref.	
DOX-resistant breast cancer cell lines. MCF7 and MDA-MB-231	Lnc RNA-H19		Unknown			BC	DOX resistance		Inhibition of apoptosis and enhancing of cell proliferation and drug resistance		(55)	
ER-positive BC cells	LncRNA-UCA1		Cleaved Caspase 3			BC	TAM resistance		Caspase 3 intracellular levels are decreased impairing TAM-induced apoptosis		(56)	
HER2-positive BC cells	LncRNA-SNHG14		Bcl2/BAX signaling pathway			BC	Trastuzumab resistance		LncRNA-SNHG14 may induce resistance to trastuzumab through inhibition of Bcl2/Bax apoptotic pathway.		(57)	
Eca109 MDR cells	linc-VLDLR		Eca 109			Esophageal cancer	MDR		Linc-VLDLR EVs, secreted by the drug-resistant esophageal carcinoma cells, could cause the acquired drug-resistance phenotype of target cells by regulating the expression of ABCG2		(58)	
Sunitinib-resistant renal cancer cells	LncARSR		Endothelial cells			Renal cancer	Sunitinib		LncARSR is identified as a mediator of sunitinib resistance in renal cell carcinoma by acting as a competing endogenous RNA for miR-34 and miR-449, and show that small EV-mediated transmission of lncARSR can confer resistance to sensitive cells		(59)

**Table:** 

Other cargoes												
Cell of origin		EV content		Target	Cancer type		Type of resistance		Mechanism		Ref.	
Cervical cancer cells		ceRNA of miR-34b		Unknown	Cervical cancer		CPT resistance		EVs carrying HNF1A-AS1 as a ceRNA of miR-34b to promote the expression of TUFT1 and the drug resistance of CC cells		(60)	
Mouse mammary tumor TS/A cells		Unknown		Unknown	BC		Immunosuppression		Inhibition of NK cell tumor toxicity stimulated by IL-2		(61)	
Metastatic BC cells		Unknown		Unknown	BC		Immunosuppression		Blocking T-cell proliferation and NK cell cytotoxicity		(62)	
DOX-resistant MCF-7 cells		DOX		N/A	BC		DOX resistance		DOX accumulation in shed vesicles		(63)	
TAM- and metformin-resistant MCF-7 cells		N/A		N/A	BC		TAM and metformin resistance		ERα decreased activity. Activation of AKT and AP-1, NF-kB, and SNAIL1		(64)	
Patients with mBC resistant to hormonal therapy		mtDNA		N/A	BC		Endocrine therapy resistance		Promoting ER-independent oxidative phosphorylation		(65)	

ADM, adriamycin; BC, breast cancer; CPT, cisplatin; CRC, colorectal cancer; DOC, docetaxel; DOX, doxorubicina; EPI, epirubicina; GEM, gemcitabine; MDR, multidrug resistance; PTX, paclitaxel; TAM, tamoxifene; N/A, not applicable.

The term EVs used in this review, independent of the term used in the article referred to, refers to a mixed population of small EVs ranging from 30 to 200 nm since the available isolation methods are not able to discriminate vesicles originated from different pathways.

## EVs mediated drug resistance

The hallmarks of drug resistance are basically summarized in six points: (1) alteration of drug targets (2), activation of drug pumps, (3) detoxification mechanisms, (4) reduced susceptibility to apoptosis, (5) increased ability to repair DNA damage, and (6) altered proliferation. Also, local modifications of stroma, tumor microenvironment (TME), and local immunity could contribute to the development of resistance (66). Keeping in mind these notions, EVs are involved in cell–cell communication and cargo sharing/delivery, and these characteristics have been associated with chemo- and targeted therapies’ resistance as detailed here. In the next paragraphs, the most important mechanisms by which EVs regulate drug resistance will be described.

### Activation of drug-efflux pumps

Efflux pump mechanisms are physiologically important in many processes such as toxin clearance from the gastrointestinal tract, elimination of bile from the hepatocytes, effective functioning of the blood–brain and placental barrier, and the renal excretion of drugs. In drug-resistant tumors, the overexpression of these proteins (67) allow the cells to reduce the intracellular drug concentration to a sublethal dose. Many research papers described the role of EVs in the transferring of drug efflux pumps from resistant to sensitive cancer cells. Among the delivered proteins are frequently described ATP-binding cassette (ABC) family, like P-glycoprotein (P-gp, MDR1, and ABCB1), breast cancer (BC)-resistant proteins (ABCG2, BRCP, and ABCA3), and multidrug-resistant protein 1 (MRP-1) (45, 46, 68–72). The mechanism by which EVs transfer proteins among cells is commonly called EVs-mediated horizontal transfer of drug efflux pumps. BC cells were able to export doxorubicin in the extracellular medium by EVs shedding, thus reducing intracellular accumulation of the drug. Moreover, EVs mediate the transfer of functional proteins or RNAs (miRNA and mRNA) that modulate the expression and function of P-gp. The P-gp is found to be overproduced in cancer cells to remove cytotoxic drugs from cells and is demonstrated to cover a pivotal role in drug resistance together with TRPC (transient receptor potential channel) proteins (73).

EVs could also transfer drug metabolizing enzymes to inactivate drugs. Yang et al. described that the expression of GSTP1 (glutathione S-transferase P1), an enzyme belonging to phase II drug-metabolizing proteins, was higher in doxorubicin-resistant cells and in their EVs, which are capable of transferring the GSTP1 enzyme to sensitive cells (74). Accordingly, a high level of GSTP1 in circulating EVs may be an indication of a drug-resistant profile and could be used as a drug resistance predictive marker (74) as already demonstrated for the expression of GSTP1 on tumor cells (75, 76).

Cell viability could even be enhanced by EVs’ transferring of pro-survival factors like cell surface receptors, miRNAs, and cellular proteins. These cargoes could improve cell viability by decreasing apoptosis and activating proliferative signals (77–81).

### Intercellular communication between the microenvironment and tumor cells

As described in the *Introduction*, EVs are involved in cell–cell communication. This mechanism could play a role in the bidirectional crosstalk between tumoral and stromal cells also regarding drug resistance mechanisms. EVs derived from cancer-associated fibroblasts (CAFs) are described to be involved in drug resistance in different types of tumor. In colorectal cancer, EVs derived from CAFs are able to induce chemoresistance to 5-FU and oxaliplatin both *in vitro* and on patient-derived mouse xenografts (82). CAF-EVs are able to promote stemness and resistance in CRC cells *in vitro* and *in vivo* also by transferring the lncRNA H19 (83), H19 is an activator of β-catenin pathway. Previous studies demonstrated that the β-catenin pathway is involved in tumor progression and drug resistance (84–86). Interestingly, CAFs are naturally resistant to gemcitabine and their EVs transfer the gemcitabine chemoresistance phenotype in pancreatic ductal adenocarcinoma (PDAC) by delivering the SNAIL mRNA that increase SNAIL protein expression promoting proliferation and drug resistance (87). A recent work highlighted that CAF-EVs are involved in oxaliplatin resistance in CRC by transferring the CCAL (colorectal cancer-associated lncRNA) and activating the β-catenin pathway (88). CCAL interacts with mRNA-stabilizing protein HuR (human antigen R) increasing β-catenin mRNA and protein levels. Another work described the effect of stromal EVs in multiple myeloma cells inducing resistance to bortezomib, which could be linked to the activation of JNK, p38, p53, and Akt pathways (89). The release of EVs from mesenchymal stem cells carrying miR-222/miR-223 is linked to drug resistance in BC cells (90). ZEB1 mRNA encapsulated in EVs derived from mesenchymal transformed lung cells can transfer gemcitabine and cisplatin chemoresistance and the mesenchymal phenotypes to epithelial NSCLC cell line (91).

### RNA (miRNA, lncRNA, and mRNA)-mediated drug resistance

Micro RNAs are small noncoding RNAs of 13–29 nucleotides involved in gene regulation and different biological and pathological processes, including the formation and development of tumors and drug resistance. In the last years, miRNAs are one of the most studied cargoes of EVs. As described, drug resistance mechanisms are heterogeneous and complex, and most of them are also regulated by miRNAs (92). miRNAs could promote drug resistance through the activation of metabolizing enzymes, in turn favoring drug inactivation or the expression of drug efflux pumps. The transfer of miRNA-365 by tumor-associated macrophage (TAM)-derived EVs to pancreatic ductal cells is described to induce resistance to gemcitabine in pancreatic adenocarcinoma by upregulating the triphosphate-nucleotide pool in cancer cells and inducing the cytidine deaminase enzyme that is able to inactivate gemcitabine (93). As mentioned, EV miRNAs could regulate the expression of ABC transporters that are involved in the efflux of intracellular drugs. It is described that, in ovarian cancer (OC) cells, there is an inverse correlation between the expression of Caveolin 1 (Cav1) and ABCB1, and this proportion is supposed to be driven by Cav1 (94, 95). Kanlikilicer et al. demonstrated that Cav1 levels in macrophages when co-cultured with OC cells are selectively dysregulated by the release of miR-1246 *via* EVs by OC cells. miR-1246 secreted in EVs inhibits the expression of Cav1 and upregulates ABCB1 expression to induce tumor-promoting phenotype and drug resistance *in vitro* and *in vivo* (96). As described for the transport of drug efflux pumps, even miRNAs could display a double-action behavior in the occurrence and development of drug resistance. Some miRNAs could have a positive effect on drug resistance, enhancing drug sensitivity in cancer cells. An analysis conducted by Liu et al. showed that EVs containing miR-128-3p were able to downregulate the expression of the MDR5 protein thus enhancing oxaliplatin sensitivity in resistant colorectal cancer cells (97). Another way to inhibit drug resistance is the regulation of glycolysis. Cancer metastasis, invasion, and drug resistance are also dependent on the anabolic profile of tumor cells that promotes the decrease in extracellular pH leading to the reduction of cytotoxic T-cell function in the TME acquiring strong survival advantages (98, 99). The GLUT protein family is involved in the intracellular uptake of glucose (100) and the regulation of glycolysis could be a strategy to contrast drug resistance (101). GLUT1 is demonstrated to be overexpressed in several tumors (102, 103), and its activation is associated with the regulation of mTOR. A decreased expression of mir-100-5p is described to be involved in drug resistance in many tumors. mTOR is the target gene of miR-100-5p that decreases its expression, enhancing drug sensitivity in cancer cells (104).

mRNA-mediated EVs are another player in the resistance process. Cao et al. demonstrated that EVs containing the DNMT1 mRNA (DNA methyltranferase 1) induce the overexpression of this enzyme in the recipient cells, playing an important role in the cisplatin resistance mediated by EVs in the xenograft model (105). In this research work, the underlying mechanism is not investigated, but it could be speculated that the dysregulation of Wnt and PI3K/AKT/mTOR signaling pathways, caused by an altered methylation status in a variety of genes, was described to be associated with resistance to standard treatments in many types of cancer (106). It was also demonstrated that BC cells resistant to doxorubicin possess an increased level of mRNA coding for a detoxifying enzyme (GSTP1) and EVs derived from those cells are capable of transferring the mRNA to sensitive cells and inducing resistance (74). *In vitro* and *in vivo* experiments demonstrated that normal astrocytes can protect glioma cells from apoptosis induced by Temozolomide (TMZ) through the transfer of the mRNA of O-6-methylguaninene-DNA methyltransferase (MGMT) by EVs (107). EVs transfer of Zinc finger E-box homeobox 1 (ZEB1), a transcription factor involved in the epithelial-to-mesenchymal transition (EMT) process, induces the mesenchymal phenotype and drug resistance in recipient lung cancer cells (91, 108). In particular, this work described how EVs derived from mesenchymal oncogenically transformed lung cells can transfer chemoresistance and the mesenchymal phenotype to recipient cells.

LncRNA delivered by EVs often serves as competing endogenous RNA (ceRNA) to help miRNA in their drug resistance regulatory mechanisms (109). LncRNAs have been identified to be involved in cancer drug resistance by affecting the expression of drug metabolizing enzymes (110). Two studies described that EVs transferring lncRNA linc-ROR (111) and linc-VLDRLR (112) induced sorafenib and doxorubicin resistance in HepG2 cells (hepatocellular carcinoma) by activating the TGF-β pathway (111) and increasing the expression of ABCG2 (112). LncRNA urothelial carcinoma-associated 1 (UCA1) in NSCLC is associated with the modulation of a gefitinib-resistant phenotype by decreasing the expression of miR-143 and consequently increasing the expression of its target FOS-like 2 (113). LncRNA SBF2-AS1 is identified to be ceRNA of miR-151 and is involved in the mechanism of DNA repair that is one of the leading mechanisms of resistance to TMZ in neurological cancers (114). In glioblastoma patients, the presence of EVs lncRNA SBF-AS1 in the serum was found to be associated with TMZ resistance (115). LncRNA could also act by regulating some RNA-binding proteins as demonstrated for AFAP1-AS1 associated with shorter time survival of HER-2-positive BC patients linked to trastuzumab resistance. AFAP1-AS1 is responsible for trastuzumab resistance by upregulating HER-2 expression through the binding of the RNA binding protein AU-binding factor 1 (116).

## EVs and possible applications as biomarkers of tumor therapy resistance

EVs can be isolated from various types of body fluids including blood, urine, and saliva. It is demonstrated that in the cancer patient population, the amount of EVs present in the blood is more than double compared to healthy individuals (117), suggesting that they could be new biomarker candidates (118). A correlation between serum EVs containing miR-146-5p could predict the efficacy of cisplatin in NSCLC patients in advanced stages and utilized for the real-time monitoring of drug resistance manifestation (119). Xiao et al. described that EVs derived from the serum of drug-resistant CRC patients (5-FU resistance) are enriched in TAG72 (tumor-associated glycoprotein 72) (120, 121). In BC preclinical models, it is demonstrated that the cargoes of EVs are influenced by the stress induced from drugs and could be correlated to the transfer of resistance in metastatic sites mediated by the Pg-P protein (40) or by miR-423-5p (122). Leukemia-derived EVs are described to induce IL-8 release in bone marrow stromal cells, thus protecting the cells from the effects induced by chemotherapy (123). A high level of IL8 promotes the expression of Pg-P and is required for the expression of the MDR profile in BC (124) and, in renal cancer, is described to be associated with sunitinib resistance (125). According to the described implication of EVs in drug resistance, it could be useful to set up methods for rapid isolation and characterization of tumor-derived EVs to improve the personalization of the therapies and to predict the drug response of the patient. Moreover, targeted drugs against tumor-derived EVs should be studied to reduce their non-beneficial effects as described in the next paragraph.

## Targeting EVs to reduce cancer chemoresistance

Considering the importance of EVs in the regulation of chemoresistance, a few drugs were utilized to inhibit their production, release, or action.

It is described that drug-resistant cancer cells could produce an increased number of EVs than their drug-sensitive counterpart, thus contributing to the spread of resistance (45, 126–128). Some studies reported that, in drug-resistant cells, there is a direct association between the presence of drug resistance mediators and molecules involved in the production of EVs. For instance, Annexin A3 is a protein involved in OC platinum resistance and is also demonstrated to have a role in the EVs’ production (129, 130). In the last years, an increasing number of studies investigated the possibility to inhibit the release of EVs from cancer cells. GW4869 is an inhibitor of the neutral sphingomyelinase (131) and is able to sensitize cisplatin-resistant OC cells by reducing EVs trafficking (105). Moreover, rhamnose-emodin is a molecule that is described to reduce the secretion of EVs from doxorubicin-resistant BC cells, thus reducing the expression of EVs miRNAs involved in chemoresistance (132). Therapeutic targeted antibodies against cell surface receptors may be neutralized by EVs interaction. Aung et al.’s research group described how Rituximab (anti-CD20) is quenched by EVs expressing the target protein. The authors also demonstrate that by blocking EVs biogenesis with indomethacin, the therapeutic benefits of the therapy were restored (45). In another work, indomethacin is used to block EVs secretion in order to increase the amount of cytoplasmatic doxorubicin, its accumulation in the nucleus, and cytotoxicity (133). In a CRC model, it was demonstrated that the interaction mediated by EVs between cancer stem cells and fibroblasts promoted the resistance to 5-FU and oxaliplatin and can be reverted by blocking the release of EVs (82). Xie et al. developed functionalized silica mesoporous nanoparticles (NPs) able to selectively bind EGFR^+^-EVs derived from NSCLC through aptamer recognition. NPs, after binding to EVs in the bloodstream, are delivered to the liver and excreted in the intestinal tract to be removed from the organism. It was demonstrated that by employing this system, the *in vivo* cancer metastatic overgrowth could be reduced (134).

## Conclusions

The discovery of new cancer therapies is a stimulating topic that is investigated by a lot of researchers all over the world. The need for new therapeutic approaches is required because of the interpatient variability in terms of drug response, and also the development of drug resistance represents a very hard hurdle to overcome. Drug resistance appears in almost all types of cancer, and the underlying mechanisms are not yet clearly understood. In the last few years, the wide implication of EVs in drug resistance has been investigated, and in this manuscript, the major implications in this process are described and summarized. Although many described experiments are limited to preclinical and often to an *in vitro* stage, it is necessary to deeply investigate the roles of EVs in cancer drug resistance for many important aspects.

First of all, the involvement of EVs in drug resistance and their profiling could be exploited in the clinical approach to define new hallmarks of prognosis of drug response avoiding invasive procedures. On the other hand, as already explained, clarifying the role of EVs on drug resistance could stimulate the development of new anti-cancer strategies based on EVs targeting to revert drug resistance. Most importantly, cancer-released EVs should be deeply characterized, and their peculiar properties should be investigated. In this way, the development of new targeted strategies able to discriminate tumor-derived EVs could be set up. Moreover, the employment of artificial EVs could be considered in order to revert drug sensitivity (135, 136). EVs have already been described to possess very suitable properties as DDS to be loaded with different cargoes (drugs, miRNA, and proteins) displaying high biocompatibility, and the capacity to target cells is 10 times higher compared to liposomes of the same size (137–140). EVs could also represent a new DDS against neurological malignancies due to their ability to cross the blood–brain barrier (141, 142). Due to the possibility to produce engineered EVs *in vitro*, they could be developed to target different types of malignancies. There is also the possibility of studying artificial EVs for therapeutic employment with the advantage of producing standardized EVs with a defined content to facilitate the transition into a clinical application.

## Author contributions

SP wrote and revised the text. FR and VC conceptualized the work and edit the final version. All authors contributed to the article and approved the submitted version.

## Funding

This research was funded by Italian Ministry of Health – Ricerca Corrente.

## Acknowledgments

We thank all the colleagues from “Aviano Centro di Riferimento Oncologico” and from Ca’ Foscari University for the constructive discussions about the topic of the review helping us to develop the final work.

## Conflict of interest

The authors declare that the research was conducted in the absence of any commercial or financial relationships that could be construed as a potential conflict of interest.

## Publisher’s note

All claims expressed in this article are solely those of the authors and do not necessarily represent those of their affiliated organizations, or those of the publisher, the editors and the reviewers. Any product that may be evaluated in this article, or claim that may be made by its manufacturer, is not guaranteed or endorsed by the publisher.
