# Supplementary figures and images for: The history of small extracellular vesicles and their implication in cancer drug resistance
Source: Front Oncol. 2022 Aug 24;12:948843. doi: 10.3389/fonc.2022.948843 (PMC9451101; doi:10.3389/fonc.2022.948843)

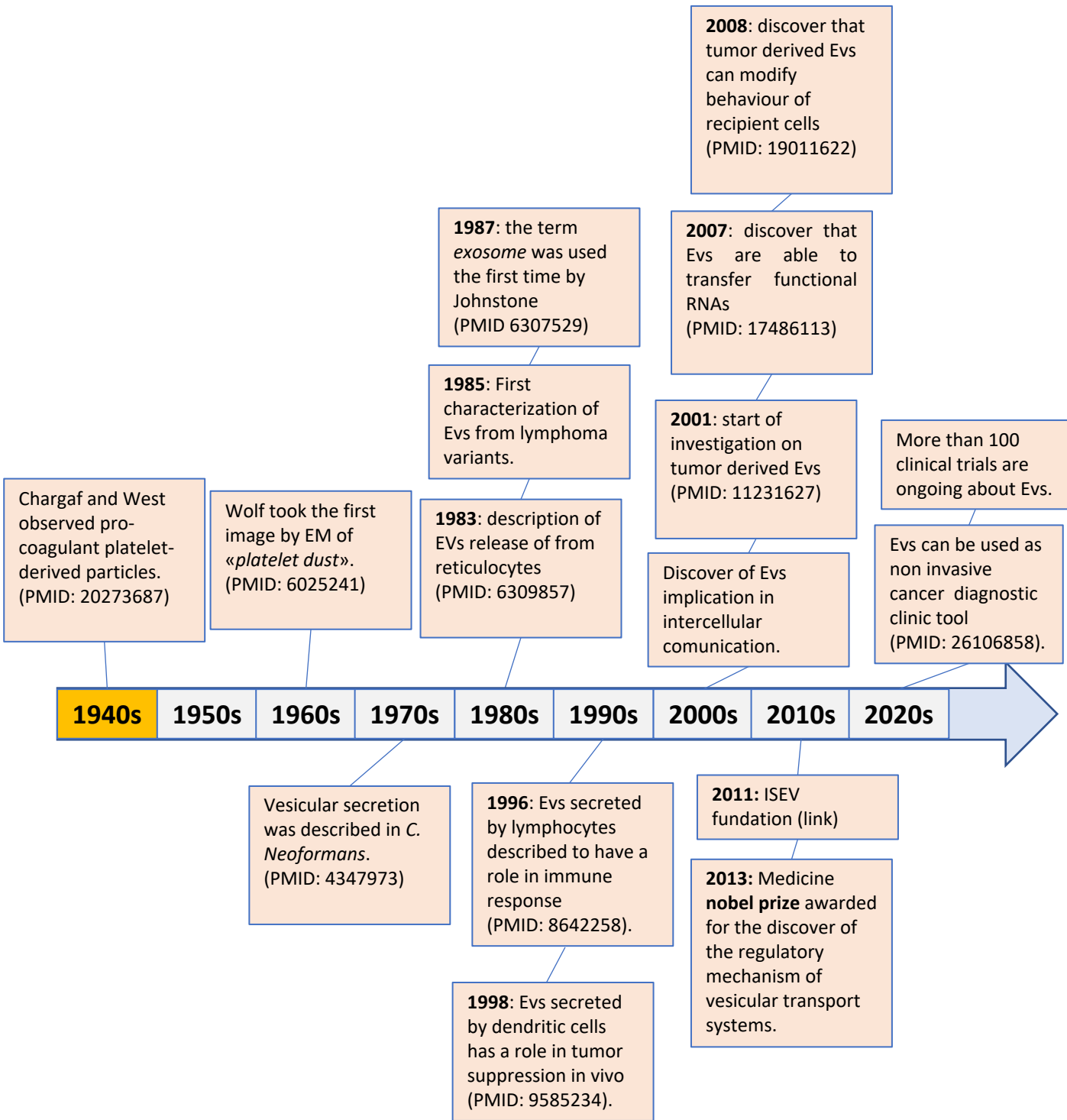

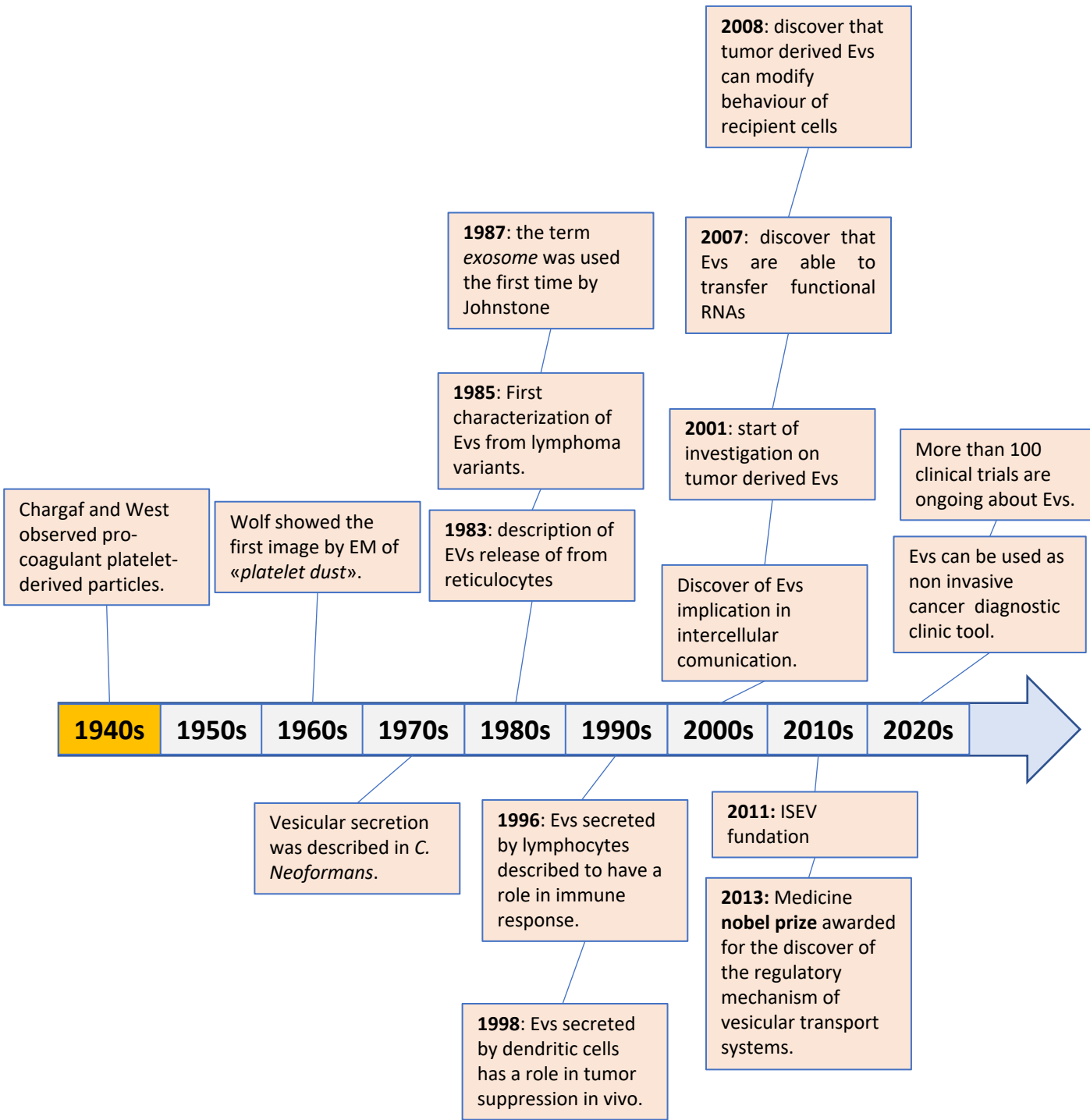

Supplement: Supplementary file 1 [file DataSheet_1.pdf]
